# A Phenomenological Psychology Study of University Teachers' Lived Experience of Being Pedagogical in Neoliberalism

**DOI:** 10.3389/fpsyg.2022.895635

**Published:** 2022-07-22

**Authors:** Yulong Li, Xiaojing Liu

**Affiliations:** ^1^School of Education, University of Glasgow, Glasgow, United Kingdom; ^2^Center for Educational Science and Technology, Beijing Normal University, Beijing, China

**Keywords:** phenomenological psychology, pedagogical tact, lived experience, casino capitalism, neoliberalism

## Abstract

Against a background of promoting the industrialization of higher education in Macau due to COVID-19's damage to the territory's major industry—gambling, the present study adopted phenomenological psychology to explore teachers' lived experience of being pedagogical in a university with a neoliberal vision and mission. Using a general structure, the findings revealed that teachers encountered challenges being pedagogical. These challenges emerged not only due to the university's corporate management, but more importantly because of a shift in perceptions—where students became like customers and teachers became self-interested—which made pedagogical relationships difficult to establish. Furthermore, teachers were found to develop negative emotions when their pedagogical actions or intentions conflicted with neoliberalism. The findings suggest that pedagogy in higher education is being challenged and transformed.

## Introduction

Susan Strange (1997, p. 1) created the term, “casino capitalism,” to convey how the economic system in the Western world has been operating like a casino: “Some of the players—banks especially—play with very large stakes. There are also many quite small operators. There are tipsters, too, selling advice, and peddlers of systems to the gullible.” Strange (1997) used the term metaphorically, but in Macau, casino capitalism has a literal meaning. On the 20th of December 1999, Macau's sovereignty was handed from the Portuguese over to the People's Republic of China (PRC) and it has been a Special Administrative Region (SAR) of the PRC ever since. Following the leadership of Beijing, the Macau SAR government has adopted a unique economic strategy of decolonization accelerating the gaming industry by breaking up the former Macau Tourism and Entertainment society's exclusive monopoly on gaming and opening it up to international and regional casino companies (Shi and Liu, 2014).

The gaming industry in Macau has increased the newly founded SAR government's credibility among the Macanese (Lo, 2009) because the casino hotels and amusement parks on the Cotai Strip, built on reclaimed land, fulfilled the local people's long held expectations of modernization (Shi and Liu, 2014) making the city appear more modern. The gaming industry also quickly made Macau one of the wealthiest economies in the world per capita (Sheng, 2016), although as the major employer and the biggest taxpayer, the gaming industry caused Macau to become excessively reliant on it (Lo, 2009; Sheng, 2016). Shi and Liu (2014) revealed that the process of decolonization in Macau is intertwined with neoliberalism. Together with the expansion of casinos, consumerism began to permeate the mentality of local people, particularly the younger generation (Shi and Liu, 2014). Giroux (2014) claimed that casino capitalism can bring about a gambler mindset of chasing self-interests and pursuing extreme individualism. As a result, many Macanese began to admire and seek luxurious products and lifestyles and thus chose to work in casinos for handsome salaries (Shi and Liu, 2014). Meanwhile, scholars noted an increase in the number of students dropping out of school to work in casinos which was coupled with teacher attrition (Morrison, 2009; Yu, 2015; Wu and Vong, 2017). Commenting on this situation, Shi and Liu (2014, p. 930) observed that everybody's “daily experiences, emotions and desires [were] associated with the expansion of the gambling sectors.” Thus, triggered by casino capitalism, neoliberalism became entrenched into Macanese society.

By monopolizing Macau's economy, the gaming industry has made the city vulnerable to shocks to tourism (Lo, 2009), as most punters come from outside Macau. Since 2020, COVID-19 has been disastrous for Macau's gaming industry and in turn many citizens' job security; however, the virus roused the SAR government to look for ways to diversify the economy. Unfortunately, over the years, the gaming industry had absorbed many school leavers resulting in a shortage of local talent with higher educational qualifications to support the city's sustainable development (Lau and Yuen, 2014). Reflecting on the situation, Ho Iat Seng, the Chief Executive of the SAR government, in the Macau Policy Address 2021, announced the city would promote the industrialization of higher education (Lin, 2021). In this way, the industrialization of higher education in Macau may force local universities to become more business- and market-oriented to improve their bottom lines (Liu, 2021).

While this transition may relieve Macau's over reliance on the gaming industry, it may inevitably have some side effects on local higher educational institutes as neoliberal reforms have brought to universities elsewhere. Long before the 2021 Policy Address, many pre-tertiary schools in Macau had implemented neoliberal changes, and a few local scholars have revealed how teaching, management, courses, and teacher identity have been impacted (see Huang and Vong, 2016, 2018; Huang, 2018). Although the voices of Macanese educators who have spoken out against neoliberalism have mostly been drowned out by those who are enthusiastic about educational marketization, fortunately, the studies of the former have revealed that the neoliberal discourse has not completely occupied education in Macau (Huang and Vong, 2016, 2018), reserving some space for negotiation and reflection. Nevertheless, there are few studies on Macanese university teachers' experiences and perceptions of neoliberalism; accordingly, the present study is not only exploratory but also reflective in the face of the enthusiasm for the industrialization of higher education.

## Literature Review

### Neoliberalism

Neoliberalism, as a contemporary school of thought and an antidote to the crisis that threatens late capitalism, was originally conceptualized by the Mont Pelerin Society, composed of scholars like Friedrich von Hayek, Milton Friedman, and Karl Popper in the 1940s (Harvey, 2005). They inherited thoughts from traditional liberalism and neoclassic economics, firmly believing in private property and free, competitive markets as the protectors of freedom of thought and expression; meanwhile, they warned that arbitrary power is expanding and invading such freedom in the West (Harvey, 2005). After WWII, while recuperating from the war, many countries kept their national strategies aligned with Keynesian ideals, meaning state power should guarantee “full employment, economic growth, and the welfare of its citizens, and that state power should be freely deployed, alongside of or, if necessary, intervening in or even substituting for market processes to achieve these ends” (Harvey, 2005, p. 10). Undeniably, Keynesian policies sustained domestic stability and economic growth in many Western countries from the 1950s to 1960s; however, this mode later caused economic stagnation and financial crises (Harvey, 2005). Neoliberalism was considered an alternative strategy, while the actual practice of it began in the 1970s when many countries, such as the UK, the US, Sweden, India, Chile, turned to neoliberalism, and even China did so after its opening-up policy was introduced in 1978 (Harvey, 2005). Harvey (2005, p. 23) summarized how neoliberalism evolved as an alternative to the Keynesian approach:

This entails confronting trade union power, attacking all forms of social solidarity that hindered competitive flexibility, dismantling or rolling back the commitments of the welfare state, the privatization of public enterprises, reducing taxes, encouraging entrepreneurial initiatives, and creating a favorable business climate to induce a strong inflow of foreign investment.

Simply speaking, neoliberalism restricts the intervention of government into many societal aspects, while allowing corporate values and competitive market principles into all public domains. Indeed, neoliberalism to some extent solves the problem of economic stagnancy; however, it can also sabotage public goods, for example, higher education (Giroux, 2002). In this way, universities become like companies, and its products are not actual items but knowledge economy-driven technologies, skills and designs (Olssen and Peters, 2005) and highly skilled labor (Becker, 2006). Neoliberalism has brought corporate values like marketization into universities, and it has also transplanted corporate management techniques, like managerialism, standardization, accountability, and performativity into tertiary education (Giroux, 2002; Olssen and Peters, 2005; Apple, 2006; Ball, 2016). In the neoliberal environment, university teachers also face increasing career instability (Enslin and Hedge, 2019), hierarchical auditing supervision (Olssen and Peters, 2005), decreasing of tenured positions (Giroux, 2002), and eroded authority in faculty decisions, which has created extra work for them (Seeber and Berg, 2016).

### Pedagogy in the Time of Neoliberalism

The meaning of education is inevitably influenced by neoliberalism. Van Manen (1991) expressed his concern that pedagogy itself is also threatened by neoliberalism, which standardizes many aspects of classroom teaching, reducing the skills, competence, and professionalism of instructors. This uncertain situation requires teachers to reflect but also to be ready to criticize (Van Manen, 1991, 2015). Pedagogy is a lived experience of adults; it is “both the tactful ethical practice of our actions as well as the doubting, questioning, and reflecting on our actions and practices” (Van Manen, 2015, p. 33). Adults who are pedagogical are those who are always reflecting about distinguishing what is good for their children to facilitate their “positive being and becoming” (Van Manen, 1991, p. 18). Van Manen (1991, p. 5) stressed pedagogically oriented teachers should be students' “in loco parentis,” offering care, guidance, and protection to students just as their parents did or even exceeding what their parents did. Teachers need pedagogical tact to become in loco parentis; they should intuitively understand their students; they should know how and when to give guidance while not being hypocritical and selfish (Van Manen, 1991). Pedagogical tact can only be obtained through praxis, as it comes only from teachers' continuous reflection-in-action, rather than following fixed instructions in planned situations (Van Manen, 1991). Preserving the tradition of pedagogy is beneficial to students and society in the long run because people's pedagogical narratives can be passed along over generations. Van Manen (2015) found that the pedagogical moments people experience unconsciously influence them for the rest of their lives. Pedagogy should be a stronghold that adults build to safeguard what is valued, i.e., childhood, love, and ethics, against external powers like neoliberalism (Sandvik, 2020). However, neoliberalism poses challenges to teachers. Generally, phenomena embedded in neoliberalism can damage university teachers' pedagogical relationship and obligation toward their students. Accordingly, knowing how teachers in a neoliberal university perceive or perform pedagogy appears warranted.

### The University's Neoliberal Agenda

The university we focus on in this study is a private institution. Although it has never publicly acknowledged its neoliberal goals, the university's owners have decided on a corporate way of management. Echoing the Macau Policy Address 2021, the university released a new vision and mission i.e., to provide services for the region, to nurture application-oriented talent, to increase student recruitment, and to operate under the disciplines of under market-oriented principals. Thus, the language it uses to describe its vision and mission represents its evolving neoliberal nature (see Allatt and Tett, 2018), which therefore, presents a suitable venue for investigating Macanese university teachers' lived experience in neoliberalism.

Studies in Macau have investigated how primary school teachers (e.g., Huang and Vong, 2016) and middle school teachers (e.g., Huang and Vong, 2018) have resisted neoliberalism in their teaching practices. Despite the prevalence of neoliberalism in their respective working contexts, these studies have shown that the neoliberal discourse in education remains incomplete reserving space for teachers to resist. Such space also becomes a rationale for us to position our study. Further, unlike the present study, their research followed the tradition of post-structuralism rather than a phenomenological psychology approach. Therefore, the present study aims to answer: What are the university teachers' lived experience of being pedagogical in neoliberalism?

## Research Methodology

Because the present study concerns ordinary people's directly lived mundane life in context (Englander and Morley, 2021), we chose phenomenological psychology as the research methodology to uncover university teachers' lived experience in neoliberalism. Phenomenological psychology was founded by Giorgi (1970) in the 1970s based on his critical reflection of the positivist-led modern psychology, which Giorgi (in Englander and Morley, 2021) critiqued because it was inadequate for studying human behavior. Beginning from Husserl's (1977) transcendental phenomenological philosophy, Giorgi (1970) combined psychological attitudes into Husserl's philosophical phenomenological process of description, reduction, and essence (Giorgi et al., 2017).

Regarding the modified process of description, phenomenological psychology permits a third person, or research participant, to use naive or non-phenomenological language to describe their lived experience (Giorgi et al., 2017), which is distinct from many other phenomenological methods, like those in Van Manen (1990), which obtains data by cultivating the research participants to produce their first person's text. The modified reduction in phenomenological psychology avoids restraining research participants' subjective consciousness, as this methodology promotes humans as “being in the world” (Giorgi et al., 2017, p. 181), who have direct consciousness about their lived world (Englander and Morley, 2021). Unlike philosophical phenomenology, which aims at universal essence, phenomenological psychology can reach the typical essence based on structures derived from each case that can be compared with results from similar studies (Giorgi et al., 2017). Thus, in planning the research, we followed the five step data analysis model of Giorgi et al. (2017):

(1) “Initial reading for a sense of the whole”; (2) “Adopting the phenomenological psychological attitude”; (3) “Dividing data onto meaning units”; (4) “Transformation of everyday expression to psychological meaning”; and (5) “Returning to the whole and moving toward the general structure” (Englander and Morley, 2021, p. 7–12).

We also sought important suggestions from Englander and Morley (2021) while conducting the analysis.

We invited five teachers for semi-structure interviews to discuss their pedagogical stories. Although written anecdotes are often used in phenomenological research (Van Manen, 1990), we chose to interview them because research participants tend to produce more description than written ones (Giorgi et al., 2017). Phenomenological psychology's focus on people's lived world reminded us to choose a data collection protocol that fits with the participants' daily customs (Englander and Morley, 2021), and as the teacher participants in this research were used to delivering lectures and providing verbal explanations, we thought the interview would be more suitable and natural. After we made appointments with them, we sent the discussion items to them along with informed-consent forms to ensure they were aware of the topics and the voice-recording. As universities in Macau had resumed face-to-face teaching during the time of the interviews, all the participants were interviewed in person with face masks on. All the interviews were conducted in Mandarin Chinese, the working language of the fieldwork university. The second author, fluent in English and Chinese, transcribed the interviews verbatim and translated them into English. However, only four interviews remained for use as one interview was discarded because it did not contain adequate content for our purposes. According to Giorgi et al. (2017, p. 183), “inadequate ones [descriptions] [that] do not yield significant outcomes … are usually discarded.”

The lead author read the translated documents thoroughly and reflexively to have an initial understanding. He then took a phenomenological stance to highlight the meaning units for each participant. After changing the first person to the third person in each meaning unit of the transcripts, he pasted the near-original language of each meaning unit in succession in the 1st column of a table, according to the methods advised by the experts, i.e., psychological reduction (Englander and Morley, 2021). In doing so, the lead author finished the psychological epoche of the participants' descriptions by putting his own interpretations in parentheses (Englander and Morley, 2021). Then the second author reviewed the descriptions mirroring the meaning units in the 1st column. We then added another two columns (2nd and 3rd) in parallel with the first column to, respectively, refer to our phenomenological and further elucidation of the meaning units. Englander and Morley (2021) pointed out that the 2nd and 3rd columns are for researchers to elucidate rather than to pamper the psychological meaning from the 1st column to help the participants' everyday consciousness emerging into generalisable psychological relevance. Although Giorgi et al. and his colleagues suggested only two tiers, i.e., columns (e.g., Giorgi et al., 2017), we are more in favor of what Englander and Morley (2021) advised, that is, to extend the two-tier column into three or even more for the sake of crystallizing the lead author's analysis process because the second author in the present study needed to review and validate them. Table 1 shows an excerpt of the whole table (see Appendix in Supplementary Material) as an example.

**Table 1:** An example of psychological reduction in the three-column table, Meaning Units equals MU, and P1 means Participant 1.

Column 1: The participants' natural expressions	Column 2: The researchers' phenomenological psychological elucidation	Column 3: The researchers' further elucidation
MU 1 of P1: P1 has such a heavy workload, and there are so many students; therefore, she often feels very tired.	P1 often feels fatigue as she faces a big workload and large numbers of students.	The teaching load and large class size makes P1 fatigued.
MU2 of P1: P1 thinks she is a teacher, and she has a close relationship with students over a period of time, such as a semester. But this kind of evaluation (students judging teachers' teaching) is harmful to the relationship.	P1 believes that student evaluation of teaching (SET) sabotages what should have been a close teacher and student relationship.	P1 thinks students evaluating teachers becomes a barrier in her relationship with students.

After the second author's validation, the lead author transformed and organized all the elucidated psychological aspects from the 2nd and 3rd columns and developed situated structures (Englander and Morley, 2021) for each participant. Separating situated structures is factitive in reaching to the psychologically generalisable essence in the later general structure (Englander and Morley, 2021). Finally, after the two authors discussed, the lead author used an eidetic reduction attitude to discard the language which potentially disturbed the rigor of the general structure to make it internally cohesive, interdependent, and easy to demonstrate the psychologically generalisable essence of the participants' lived experience (Giorgi et al., 2017; Englander and Morley, 2021).

## Findings

### Situated Structures of the Participants

P1 worried that the situation in the world today is causing students to be more utilitarian, demotivating them, and making them inactive or perfunctory in their studies. Out of her ethos as a teacher, P1 explained that it is important to intervene and challenge the students by raising her course requirements. Although she sometimes explains her good intentions to her students and attempts to lead them to think more about their future, the students often misunderstand her. However, what makes P1 upset is that her university deploys a student-evaluating-teaching system (SET), through which some students write negative feedback on her teaching in retaliation for her pedagogical intervention. The SET system has created a conundrum for her. On the one hand, she wants to provide a good education for her students, which sometimes requires being strict, but on the other hand, if she receives too much negative feedback (as a result of her strictness), her annual review result may be unsatisfactory. Eventually P1 felt that her predicament sabotaged her relationship with the students, not to mention her teaching and wellbeing. Further, the university provided little support for her, and this coupled with a heavy workload led to her being burnt out. Her experience also made her question the real meaning of higher education in her institute.

P2 believed being strict to students is important for their learning. She enjoys giving advice to students as her way of being pedagogical. However, both of her pedagogical postures encountered challenges. P2's university has a stringent set of guidelines regarding teachers' classroom performance using the SET system, which means it is easy to incur students' complaints or negative evaluation if P2 irritates them. P2 shared that she once received negative and disingenuous comments from her students. From then on, she stopped being strict and instead turned to pleasing students, although she is aware that this spoiled them. For fear of being criticized by students and out of a mindset of being unsatisfied toward the university's way of disciplining, P2 now only gives advice to students who voluntarily approach her for help after class rather than devoting the time and effort that she used to provide to all her students.

P3, once part-time, and now a full-time teacher at the university, used to enjoy teaching, and spending time on teaching preparation believing that students benefited from his classes. However, after becoming full-time, he has had to cope with more assigned errands and meetings with colleagues. However, he believes he became disillusioned when the university standardized and quantified every teachers' performance and workload; he questioned whether it is possible to compare instructors with various backgrounds who teach different disciplines. He began to worry and became distracted from advancing his teaching craft. P3 claimed that when he stopped reading as part of his teaching preparation, he stopped updating his lessons and became bored using his old materials while teaching. He also noted that he spent less time on students after becoming full-time, as he wanted to leave more time for himself.

P4 believed the best education for university students is to develop their thinking, which is at the core of their work so he kept this ethos both inside and outside of his classes. However, his heavy teaching load was so burdensome he could barely keep up with his teaching duties leaving no time for deep thinking. As a result, P4 felt he could not fully prepare his lectures, which he thought was letting his students down. P4 also mentioned that students have too many lessons to spare enough energy to learn in-depth in their chosen field. P4 felt that he was failing to provide a good quality education to his students, and sometimes he was burned out with no interest in teaching. Thus, based on his situation, P4 questioned whether his university management understood the true nature of higher education.

### General Structure

In the environment of neoliberalism, it has become more difficult for teachers to fulfill their pedagogical commitments because their pedagogical intentions are challenged by neoliberal management techniques. These techniques include SET scores that quantify teachers' performance and sometimes lead to disciplinary action. The neoliberal approach also fails to recognize or support teachers' unquantifiable contributions such as when they tutor individual students. Furthermore, teachers' pedagogical commitments also face challenges from the various entities of neoliberal discourse. These include students who are nurtured to act and think like consumers, refusing to establish a pedagogical teacher-student relationship. They also include academic colleagues who fully buy into the neoliberal discourse and enjoy competing with each other. Sometimes the entity is the university itself, which acts like an enterprise, bringing industrial production principles into higher education. Facing the tension created between pedagogical intentions and neoliberalism, teachers become fatigued, hesitant, passive, insincere, self-blaming, and anxious, which leads to job burnout.

## Discussion

As shown in the general structure, the teachers all found it difficult to be pedagogical in the neoliberal environment, which echoes the neoliberal challenges confronted by Swedish teachers in Rinne's (2020) study. From the general structure, it is apparent that some of the teachers' challenges were a result of the university's corporate management, i.e., SET scores and strict discipline. Ball (2016) observed similar managerial techniques at schools steeped in neoliberalism. Some teachers in the structure also suffered from overwork, i.e., heavy teaching loads and increased student numbers, while other teachers felt they had little time for reading, thinking, research, and updating lessons. Echoing these impediments, Giroux (2002) noted how neoliberalised universities in the US irresponsibly increased enrollment and class size to boost the universities' bottom lines with little concern about the possible shortage of full time faculty or the maintenance of teaching quality. Regarding teachers' reduced time for reading and thinking, Seeber and Berg (2016) observed that research involves thinking, and university teachers not only need enough time, but more importantly they should not be controlled by time. However, when teachers are faced with increasing demands from their university, they often feel pressed for time to conduct research, which leaves them without the mood to fully prepare their lectures or build up a rapport with students (Seeber and Berg, 2016), which is similar to the teachers' experience in the present study. Dufour (2014) noted that thinking, as the basis of research, requires time and silence, and if teachers' time to think and conduct research is denied, their university's research prospects may decline.

Management style may only be a symptom, however. What lies underneath is the ontological change of the university from being a public good to a corporation; the management style described by the teachers in this study resembles what is found in industry. Aronowitz (2000) labeled this corporate approach to higher education the “university knowledge factory” while Li et al. (2021) call it a “human capital incubator.” This change of the university's nature alters stakeholders' relationships placing pedagogy as the lowest priority. Teachers' pedagogical commitments are replaced by bullet points of practice listed in contracts, and teachers' responsibility to students are now temporary and conditional (Seeber and Berg, 2016). As shown in the general structure, some of the participant teachers chose to neglect their students and resist cooperating with colleagues and management in favor of having more time for themselves. In doing so, Enslin and Hedge (2019) found teachers acted as individualistic entrepreneurs, busy with exhibiting, promoting, and pitching their excellence and cutting-edge academic achievements while competing with colleagues. They also found teachers in such a corporate environment may develop a perfunctory attitude toward teaching and their students (Enslin and Hedge, 2019). Students' changing identity has also become a challenge. Some of the teachers expressed concerns about having difficulty to build relationships with students who were like cunning customers. For example, Doherty (2007) and Seeber and Berg (2016) found students are nurtured as clients in neoliberal universities. Saunders and Kolek (2017) showed that some students in US universities identified themselves as consumers. The teachers in the study also had negative feelings when their pedagogical intentions or behavior clashed with neoliberalism, which resulted in fatigue, anxiety, self-blame, and job burnout. Like many other teachers in and beyond Macau, neoliberalism has been emotionally damaging (e.g., Huang and Vong, 2016, 2018; Dugas et al., 2018, 2020; Li and Liu, 2020; Li et al., 2021). As Van Manen (1991) observed over three decades ago, pedagogy is threatened by neoliberalism and clearly it continues to be.

## Conclusion

Against the background of COVID-19 and the gaming industry in Macau, this study aims to provide perspective on higher education's accelerating industrialization in Macau. By exploring some university teachers' lived experience of being pedagogical using phenomenological psychology, the study produces a general structure that shows teachers challenges in being pedagogical in a neoliberal environment. These challenges are not only from the university's corporate management, but equally importantly from the change where students view themselves as customers which has led teachers to become self-interested impeding pedagogical relationships with students. Teachers also experienced negative emotions when their pedagogical actions or intentions conflicted with the neoliberalism. This result is consistent with many other studies that report changes that have occurred in education in and beyond Macau upon the arrival of neoliberalism. Thus, it is hoped that the study can draw attention to how industrialization is changing the face of Macau's higher education.

## Data Availability Statement

The original contributions presented in the study are included in the article/supplementary files, further inquiries can be directed to the corresponding authors.

## Ethics Statement

The studies involving human participants were reviewed and approved by Beijing Normal University. The patients/participants provided their written informed consent to participate in this study.

## Author Contributions

YL was responsible for theoretical conceptualisation, phenomenological analysis and essay writing. XL was responsible for data collection, revision, interview translation and project application and management.

## Funding

This study is funded by Guangdong Planning Office of Philosophy and Social Science (GD21YJY08).

## Conflict of Interest

The authors declare that the research was conducted in the absence of any commercial or financial relationships that could be construed as a potential conflict of interest.

## Publisher's Note

All claims expressed in this article are solely those of the authors and do not necessarily represent those of their affiliated organizations, or those of the publisher, the editors and the reviewers. Any product that may be evaluated in this article, or claim that may be made by its manufacturer, is not guaranteed or endorsed by the publisher.
