# Supplementary material for: A Phenomenological Psychology Study of University Teachers' Lived Experience of Being Pedagogical in Neoliberalism
Source: Front Psychol. 2022 Jul 22;13:895635. doi: 10.3389/fpsyg.2022.895635 (PMC9354741; doi:10.3389/fpsyg.2022.895635)
Supplement: Supplementary file 1 [file Table_1.docx]

**Appendix.**  The three column table of data analysis

| Colum 1  The participants’ natural expressions | Colum 2  The researchers’ phenomenological elucidation | Colum 3  The researchers’ further elucidation |
| --- | --- | --- |
| MU 1 of P1:  P1 has such a heavy workload, and there are so many students: therefore she often feels very tired. | P1 often feels fatigued as she faces a big workload and large student numbers. | The teaching load and increasing class size makes P1 fatigued. |
| MU2 of P1  P1 thinks she is a teacher, and she has a close relationship with students in a period of time, such as in a semester. But this kind of evaluation (students judge teachers' teaching) is harmful to the relationship. | P1 believes that student evaluation of teaching (SET) sabotages the-should-have-been close teacher and student relationship. | P1 thinks students evaluating teachers has become a barrier between her relationship with the students. |
| MU3 of P1  P1 considers it (teaching evaluation) only as a number, and it can’t record more issues, for example, her instructional design. It can’t record how P1 cares about students. As it can’t record all of these, sometimes P1 feels a little bit unhappy. That is to say, well, we always have to help our students to develop. | P1 suspects the wholesomeness of SET. She is upset that SET only concerns numbers, omitting her pedagogical design, her deep care for students. She believes teachers should help students to develop through teaching, in contrast to the inhumane purpose of SET. | Students evaluating teachers is single-sided as it cannot reflect P1’s pedagogy in a holistic manner. |
| MU4 of P1  P1 is not trying to please them (students). However, she thinks human cognition has different developmental stages. P1 often takes herself as an example to tell her students that when she was an undergraduate student, she also could not understand anything during her four years. After P1 graduated, she started to realize that her life in university was a failure. At that time, she didn’t know how to arrange her studies, how to regulate her own emotions, and she even thought about suicide, but after she graduated, she realized what she should have done in university. So what P1 saying is, undergraduate students undergo a cognitive developmental process, and as P1 used to be an undergraduate student, she understands what undergraduate students should do. P1 has told this to her students but they may not realize it. | P1 feels it is a pity that her teaching is sometimes misunderstood by the students. She understands that her students are just too young to understand her good intentions, because she was once young and naive.  P1 also stresses that despite the misunderstanding, she never trades off her way of teaching and good intentions to please her students. | P1 thinks her students do not understand her, but it is imperative for her as a teacher to share experiences. |
| MU5 of P1  According to the score of P1’s teaching evaluation, most of her students regarded her teaching positively, and only a few of them did not understand her. But P1 stresses that teachers like her must understand that students have different levels, and some of them have problems studying, because they have had inertia for a long time. They have all kinds of inertia, and they don’t have good study habits. In P1’s class, she hopes they can become more active. To become more active means they can think and participate by themselves. Maybe sometimes P1 is a little bit too strict, so that students may not feel so good about her arrangements. P1 thinks she has to take care of the majority of students. | Seeing the results of SET, P1 reflects that she may be relatively too strict and her plan may have caused some students to be dissatisfied. P1 comforts herself that the majority students are satisfied with her, and she should care of the majority.  P1 blames the minority who dislike her teaching as those who are laziness. Her original intention was to change their laziness, which was not accepted by them. | P1 worries about the inactivity of some of her students. |
| MU6 of P1  P1 knows that if teachers only talk at the front of the classroom, without discussing individually with students, it will put teachers and students into two worlds. Thus, students may feel confused easily by just sitting there and passing the course. Therefore, P1 wants to give them more challenges, if they can’t overcome it, she will criticize them. But she feels her students can’t understand this. They don’t understand why the teacher has so many requirements. The only thing they need from P1 is to leave them alone and stop bothering them. | P1 thinks some students’ expectation of teachers’ not involving them in teaching and lowering assessment standard is spoiling them.  P1 determines to give students challenges rather than spoiling them. | While worrying about the inactive students, P1 thinks it is important to challenge them rather than not doing anything. |
| MU7 of P1  This semester P1 saw the evaluation results from her students (which were not positive), and later she reflected about them. She found that although her motivation is good as she wants to help students learn more and to change their old thinking habits and behavior inertia, but she also has to see that there must be good communication. | P1 is disappointed when seeing her SET results, and she reflects that perhaps she needs more communication with students to make sure they understand her good intention. | P1 is frustrated that student evaluating teachers becomes a resisting weapon for some students against her, putting her into a dilemma. |
| MU8 of P1  P1 has been teaching graduate courses for three years. She also supervises a few graduate students each year. She treats graduate and undergraduate students differently. She has a higher expectation for graduate students, because she hopes they can learn how to do academic work at this stage. So in P1’s classes, she sometimes adds some academic knowledge, like how to conduct academic writing and reading. But many of her students don’t understand (why she adds these contents). Later she saw their feedback, saying that their teacher sometimes taught some useless things. But P1 was actually trying to give them greater care, so she hopes they can learn more about how to do academic work. But she feels they don’t understand (what she did), so she won’t teach the same content again this year. | P1 raises her standard in teaching and supervising postgraduate students. She enlarges her regular teaching through sharing academic skill, hoping to accelerate her students’ learning. Again it incurred some students’ misunderstanding and negative feedback on the SET. | P1 is upset about her students’ misunderstanding and comments on the SET. |
| MU9 of P1  Students just don't want to use English textbooks (to learn), but actually she has explained why learning with English textbooks is necessary. However, they thought English textbooks are too difficult for them, or they just don’t want to spend more time. Actually if they can immerse themselves in it (learning English textbooks), they really can learn something from it. Anyway, they need to read by themselves and put in a lot of effort. She has told them that if they put a lot of effort into learning, they will get a lot of benefits. However, people have inertia, or maybe they have too many courses, so that they don’t want to study harder in her class. | P1 is disappointed by her students’ passiveness in using English-version textbooks. Though she shows students that they grow what they sew, the students, she believes, are either lazy or busy coping with other modules. | P1 is upset with her students’ low study motivation. |
| MU10 of P1  Our young students just like everybody else in the society, they all are very utilitarian. They may think: “ why do I need to spend so much time here? It is just a class.” But she always tells students, including undergraduates, that how much you receive (from the class) is depends on how much effort you put into it”.  For P1’s graduate students, she told them that the purpose for your learning is to change your way of thinking. You should do it by yourself. If you don't do it by yourself, no matter how many lectures you listen to or how many teachers you consult, it is useless. So P1 asked them to be devoted, and this may lead to a tension, or a conflict (with students). | P1 ascribes the tension between her students and her to their utilitarian ethos, doing the coursework with least effort. | P1 blames the utilitarian ethos her students hold. |
| MU11 of P1  The biggest feeling for P1 is that teachers like her are educating people; teachers are not planting radishes or cabbages; therefore, no matter what happened, P1 believes she should always be a conscientious teacher. But the workload for her is too heavy, P1 wants to cry if she recalls the past few years of work. In those years P1 worked so hard and it made her burned out. On the one hand, she does not want to take her teaching for granted, on the other hand, she also doesn’t want to take her research work for granted. How should P1 juggle between them? The only way is to drive herself to do more. P1 begs, “please let me work less, don’t let me teach so many classes, so many courses!” Apart from teaching in the classroom, P1 also needs extra time to prepare lessons, to communicate with students. When P1 teaches 4 parallel classes each semester, she has to prepare 4 different folders. Why? Because students in different classes are different, even if they learn the same content, they are still different. They have different personalities, and ideas. As a teacher, P1 should make adjustments. She also needs to find time to do research, which is different from teaching. If it (the heavy workload) goes on like this, there will be nothing else to do except for working herself to death. She can't be an unconscionable person who is happy to sacrifice students— like some other teachers who don’t care about what and how students learn. | P1 feels overextended to keep being a teacher strictly sticking to her academic standard and conscientious towards students because her workload is heavy. Such overextension has burned her out and made her feel helpless in the face of the university’s assigned KPIs. | P1 feels burned out and helpless while the university provides little support to teachers like her who are responsible. |
| MU12 of P1  Because P1 is strict, students cannot plagiarize or have any relaxation in doing homework. At this time, if there is not enough communication with them, they will give P1 a low score (in the SET). They might say (in the evaluation) that what the teacher taught is very boring. | P1 complains SET is becoming a revenge tool for some inactive students against her rigour in teaching. |  |
| MU13 of P1  Students should also be protected for sure. They are the vulnerable group in the relationship with teachers. We need to listen to their voices. But what P1 expects is that the teaching evaluation should not only depend on students’ voices, but it also at least look at a teacher's attitude, teaching plan, content, whether the teachers’ teaching design is academic and what contents are included in the teachers’ PPT, and then how the teacher interacts with students. However, the problem is that there is no evaluation on these aspects. Teaching evaluations should not focus on one aspect, it should be comprehensive. | Confirming the necessity of SET in protecting students’ voices being heard, P1 re-emphasized SET’s deficiency in providing a holistic evaluation on teachers’ efforts. | P1 criticizes the mechanism of student assessing teachers sacrificing teachers’ benefits. |
| MU14 of P1  P1 always emphasizes that they are university teachers, not high school teachers. A university teacher should teach something that can impact students for a lifetime. Therefore, P1 thinks they need to have a certain academic thought, have some concepts, and they have to constantly update themselves. P1 thinks teachers shouldn’t only focus on teaching; teachers shouldn’t teach too much even in middle school. What the students need now is not to refill their knowledge because they have done so in their middle school. What they need now is methods, and a greater humanistic care. Society has its problems: it is endlessly utilitarian. P1 thinks teachers need to cultivate students for their future. | P1 questions the university’s position of teachers, who should not be asked to undertake so many teaching tasks. She doubts whether students can learn from having so many lessons. P1 thinks too much teaching may reduce to inculcation, which her students do not need. P1 believes students nowadays need humanity, which university teachers can only provide by updating their knowledge. | P1 doubts her university’s direction in providing higher education. |
| MU1of P2  Now I feel the academic affairs office of a university is just like a police station. It will quantify what the teacher is doing. For example, it checks whether teachers follow the syllabus to teach, and whether teachers upload their syllabus on time. If not, the office will consider this is a teaching accident, and then the recorded teachers get deductions from scores in the teaching evaluation. I feel this way is too strict for teachers to teach in their own way. Teaching in university is different from teaching in middle school. Teachers should not use cramming methods to teach; instead, they should be allowed to teach following their research direction and interest. | P2 feels that the university’s register office is like a police station. They standardize and supervise teachers’ teaching behaviors, like checking whether the teachers have uploaded teaching materials and whether they are in line with curricula. The register office is entitled to punish teachers if they fail to accomplish any required tasks. P2 thinks such a didactic mechanism prevents university teachers from teaching, because university teachers need flexibility. P2 thinks too much discipline and control makes university teaching didactic, reducing it to a discipline bounded middle school classroom. | P2 thinks discipline and quantified assessment of teaching restricts academics and higher education. |
| MU2 of P2  A student came to my office to meet me just now. He has a poor foundation in English, so he came to re-sit the English exam. He only took a pen and I used a computer to dictate. I helped him correct mistakes. He told me that he feels very confused as a English major student. He felt that his learning foundation was not good, and then he could not understand (the teaching contents) in all the classes, and he only got three or four points on each quiz. So I tried to enlighten him and asked him what he wants to do in the future. I told him that learning English needs more exercises. Cramming for exams is definitely not a good way to learn. I shared some English learning methods with him. I feel that he has no idea about what he will do in the future. He also doesn't know why he wants to be an English major, but he had chosen to learn English. I suggested that he do more oral practice when learning English, but he said that doing oral practice is a hard work. He also said that reciting English is hard work. I said that doing anything is hard, he must work hard so that he can get rewards. | P2 shares an experience of giving suggestions to a low-performance student in her spare time. She advises him to be studious in practicing in daily life rather than temporarily cramming. She also encourages him to be aware that efforts will have returns. |  |
| MU3 of P2  After class, when I am communicating with students, I stop imparting knowledge to students, instead I care more about their further life plans. After class, the communication manner between teachers and students is different, which is a one-to-one private mode. So the student may tell me their questions easily, and I provide solutions. | P2 gives suggestions to students after class. | P2’s preference to advise students after class individually reflects her unsatisfactory attitude towards disciplined and quantified classroom activities. |
| MU4 of P2  I think the students are too young and confused about their future. I thought that since I was also an English major, I encountered similar learning experiences to them. So I would like to share some tips on learning English with them to help them overcome confusion. | P2 enjoys giving students advice as someone more experienced than them. | Advising students is P2’s way of being pedagogical. |
| MU5 of P2  Actually, I like encouraging students. However, I think some administration in our school is not appropriate, such as letting students to evaluate teachers’ teaching.  Sometimes I really want to impart a lot of knowledge to students, pushing them to learn more, but the student evaluation of teaching system forced me to change my role: from being a strict teacher to someone who pleases students because the school takes this teaching evaluation very seriously. | P2 thinks encouraging students is good for them, so she is sometimes strict and pushes students. P2 believes pushing students occasionally may make them learn. However, she is afraid of students commenting on her negatively in the SET if she becomes strict and pushes them too much. | P2 believes the existence of students assessing teachers prevents her from being strict towards them; therefore, she is unable to be pedagogical. |
| MU6 of P2  For example, sometimes I want to give a low score to students as punishment because they didn't perform very well. But at that moment I have to consider if I really give students a low score, they might be hate me, and they will give me a very low score back in the teaching evaluation. So I have to try my best to please them. | In the face of the danger of being negatively evaluated in the SET, P2 chose not to irritate students by being too strict, Instead, P2 turns to please her students. |  |
| MU7 of P2  In fact, I teach them with my heart, but the comments from some students was not consistent with my real teaching performance. Such as they said I didn’t upload learning materials regularly, but actually I had already uploaded everything and they could see it. Some students said that I should become more interesting, but I really want to ask: “Do you want to have fun in a university class? Are you sure that you want me to play Zhao Benshan’ s comedy?” I am teaching them knowledge in class, and some knowledge is indeed dull and dry, am I right? Learning is also a boring process, learning should not be always interesting. This is not an acting class, I’m not suppose to act in my class. | P2 was disheartened by some of her students’ accusation of her failing to upload materials in the online teaching system. P2 stresses that she is very careful in teaching and has done all the material uploading.  P2 is also angry about her students picking on her not being funny enough, as they have written in the SET. P2 is frustrated being regarded as a comedian, and she emphasizes that experience of learning is bitter in nature. | P2 thinks students assessing the teacher challenges pedagogy and spoils students. |
| MU8 of P2  So sometimes I must adjust my teaching to please students. I make my course very simple and easy to be learned, so that students will learn very happily and they also can get a high score. However, the fact is that they didn’t learn much in-depth contents in this class at all. | Although angry, to keep herself from the negative results in the SET, P2 chooses to compromise. So she feels she must make her students happy by making the courses easy, although she knows students will not learn in-depth knowledge in this way. | P2 chooses to please students, but she knows this is not helping them to learn. |
| MU9 of P2  If the students would like to come to my office and have a talk with me about their confusion, I’d like to talk with them and give some advice; otherwise, I would not do this because the number of students is too large, and as a university teacher I have to do research. There are also many administrative duties that can make me feel stressful. So I don’t have time to talk with students one by one. I don’t have this responsibility because the teaching affairs office in our school does not acknowledge my hard work in these aspects. They only care about the students’ comments. | P2 declares why she prefers students approaching her individually after class. She explains that she chooses to be so passive in this regard as she is very busy with accomplishing KPIs. More importantly, P2 thinks giving advice to students will not be counted by the university. P2’s mentioning of the register office only being concerned with the results in the SET demonstrates her dissatisfaction towards how the university evaluates teachers. | The university’s recognition of quantifiable production of teachers makes P2 reluctant to advise students after class, as this pedagogical contribution cannot be counted. |
| MU1 of P3  I feel that I've changed a lot. Because when I was a part-time teacher in the university, the only thing that I needed to care about was to teach well, and I didn’t need to think about anything else. During that time I even had six days to prepare for the following week.  This semester I become a full-time teacher, and I feel that I have loosened the requirement for myself, I also don’t have enough time to prepare my class. | P3 used to be a part-time lecturer in the university, but at that time his only focus was teaching. P3 finds out that when he transfers to being full-time, his time for teaching preparation shrinks, which he feels worsens his situation. | P3 thinks the shortage in time makes him focus less on teaching. |
| MU2 of P3  This also made me feel uncomfortable because the lecture I’m giving here is definitely the same lecture I’ve given before with nothing new in it. So I feel bored when I teach. | P3 is dissatisfied with his teaching content as he doesn’t have enough time to prepare. | P3 has no time to update his teaching. |
| MU3 of P3  Generally speaking, students used to like to ask me something through Wechat (because P3 did not have an office when she was a part-time teacher), even in the middle of the night, and I would always immediately reply to them. But when I moved here (a new office), I didn’t tell them where I’m working. Perhaps I need some of my own time. Maybe subconsciously I thought I'd buy myself a little more time. | P3 used to respond to students’ inquiries quickly and wholeheartedly, but now he avoids students and saves himself more time. | P3 has no time for advising students. |
| MU4 of P3  I feel that my teaching is getting worse. I don’t think that I have the same attitude in teaching as before. | P3 reiterates his disappointment in his teaching. |  |
| MU5 of P3  This year, I actually worked harder than last year, and I spent a lot of time to read. Although my time is less, I still try to read more books but as I always have many errands, it is difficult to arrange my time. I feel less leisurely than before. | P3 complains that his time to read decreases as well, due to the errands he has to do. | P3 has no time to read. |
| MU6 of P3  Involution made teachers suffer from a negative mood and being mentally exhausted. No one can really calm down and immerse themselves to reading carefully. However, I think as a teacher, you should always make progress for yourselves, otherwise you will be useless. | P3 blames involution in the university causes his tiredness. He is nervous that he may not make progress if he stops reading. Such progress is what P3 thinks teachers should do. | P3 criticizes involution disturbs reading. |
| MU7 of P3  My colleagues always give me pressure, saying they are better than me, which means they have a higher SET score. But obviously, we are in different majors; how should we compare each other? You are just being standardized. But I don’t think a comparison between teachers is necessary because we are in different majors. But this really make me feel very anxious. | P3 worries about involution among colleagues, and he questions the comparability among them. | P3 criticizes involution in the university is not reasonable. |
| MU8 of P3  Anxiety makes you less productive, you know, it's a vicious cycle. When you're anxious, although you want to read a book, but you might be constantly playing on your phone. The pressure prevents me from breathing, so I had to find a way to release it, such as playing with my phone. However, the more time I play, the more pressure I have. I used to read very efficiently, but now even when I have read this book for several days, I still can’t finish my reading. This is a vicious cycle, which make you feel awful. | P3 admits that worrying reduces his efficiency in reading, which in turn makes him worry more. | P3 worries. |
| MU1 of P4  As a teacher, I feel that I am a worker on an assembly line. It is a high intensity work and I have to work for a long time. So I think that I am unable to think in many cases. Under this situation, I still want to teach my students in a way that integrates sociology, philosophy and education to help them to expand their view of the world, of the discipline, and of themselves. | P4 compares himself to a manual labour on an assembly line. He stresses his work is long and intensive, giving him almost no time to think. Against such circumstances, P4 insists his pedagogy to students, which is to enlarge their horizons and deepen their thinking. | Despite the intensity of P4’s work leaving him little time for thinking, he attempts to keep his way of pedagogy. |
| MU2 of P4  I still feel that the system in our school forces me to make prepare too many things, and sometimes this even makes me feel powerless. It means that I only can teach students limited things as I don’t have enough energy to teach them more. How much students can learn depends on themselves; as a teacher I can’t help them more. | P4’s persistence of the pedagogical approach against the heavy workload makes him feel helpless and perfunctory. In other words, he cannot implement his ideal pedagogy. | P4 becomes perfunctory in teaching due to the workload. |
| MU3 of P4  I feel that at this school, I have to teach from day to night. Actually, my students also complain that they have classes all day and they feel very tired. However, for students, they just feel tired, but for me, having classes all day means I don’t have enough time to prepare my classes very well, and I also can’t trace whether my students have made progress. They push me to use all of my time to teach, and it makes me unable to think about my course. It also makes me have less time to read, to update some new courses. | P4 and many of his students all complains they are tired with having classes all day. P4 points out that he cannot find time to even get his classes fully prepared and get his students’ problems properly solved, not to mention to read books for self improvement. | P4 is tired of teaching. |
| MU4 of P4  Students don’t have time to think, and neither do I. They also make a perfunctory effort on their homework of my class because they have too many courses and all courses have homework. | P4 thinks his students are deprived of time to think and to carefully do their homework by having too many modules to take. | P4 finds students are too busy to focus on their assignments. |
| MU5 of P4  I think if the school needs to make adjustment, the most important thing is to understand what is real learning. Can leaning be achieved by cramming knowledge? Especially for the learning in higher education, shall we provide more chances for students to learn through thinking and practice? Therefore, from my point of view, if the school wants to make changes, it should think about what real learning is. Learning is not spoon-feeding knowledge in a short time, and learning is also not completing some tasks in a short time. The school should understand that the essence of learning and teaching is a thinking process. | P4 wonders whether the management team understands the mechanism of higher education as he thinks they seem to cram students rather than encouraging them to think. | P4 is dissatisfied with the his university in terms of how it positions higher education. |
| MU5 of P4  Most of the time I feel that I no longer like to teach. I feel it wastes a lot of my time. | P4 feels he does not love teaching because his workload is too heavy. | P4 has job burnout. |
| MU6 of P4  I feel this is really a hard work, so I feel that I don’t like to teach anymore. I can’t bring new ideas to my students; I always teach them the old stuff. | P4 thinks teaching now is bitter and he is annoyed by his inability to give students good quality teaching. | P4 feels sorry for his students. |
| MU7 of P4  As I feel so exhausted, I can’t teach very well in my class. So I often apologize to my students. I tell them that this is what I can do and I have tried my best. | P4 feels sorry that his fatigue negatively influences his teaching efficacy. | P4 feels sorry about his teaching. |
